# Aminophylline suppresses stress-induced visceral hypersensitivity and defecation in irritable bowel syndrome

**DOI:** 10.1038/srep40214

**Published:** 2017-01-05

**Authors:** Teita Asano, Ken-ichiro Tanaka, Arisa Tada, Hikaru Shimamura, Rikako Tanaka, Hiroki Maruoka, Mitsuko Takenaga, Tohru Mizushima

**Affiliations:** 1 Institute of Medical Science, St. Marianna University School of Medicine, 2-16-1, Sugao, Miyamae-ku, Kawasaki 216-8512, Japan; 2 Laboratory of Bio-Analytical Chemistry, Research Institute of Pharmaceutical Sciences, Musashino University, 1-1-2αhinmachi, Nishitokyo-shi, 202-8585, Japan; 3 Faculty of Pharmacy, Keio University, 1-5-30, Shibakoen, Minato-ku, Tokyo 105-8512, Japan; 4 LTT Bio-Pharma Co., Ltd, Shiodome Building 3F, 1-2-20 Kaigan, Minato-ku, Tokyo 105-0022, Japan

## Abstract

Pharmacological therapy for irritable bowel syndrome (IBS) has not been established. In order to find candidate drugs for IBS with diarrhea (IBS-D), we screened a compound library of drugs clinically used for their ability to prevent stress-induced defecation and visceral hypersensitivity in rats. We selected the bronchodilator aminophylline from this library. Using a specific inhibitor for each subtype of adenosine receptors (ARs) and phosphodiesterases (PDEs), we found that both A_2B_ARs and PDE4 are probably mediated the inhibitory effect of aminophylline on wrap restraint stress (WRS)-induced defecation. Aminophylline suppressed maternal separation- and acetic acid administration-induced visceral hypersensitivity to colorectal distension (CRD), which was mediated by both A_2A_ARs and A_2B_ARs. We propose that aminophylline is a candidate drug for IBS-D because of its efficacy in both of stress-induced defecation and visceral hypersensitivity, as we observed here, and because it is clinically safe.

Irritable bowel syndrome (IBS) is characterized by chronic, recurrent abdominal pain and altered bowel habits (diarrhea or constipation) and is defined by symptom criteria and the absence of detectable organic disease^1^. The prevalence of IBS in the general population is remarkably high (approximately 11% of the world’s population), with the young displaying greater susceptibility^1^. Thus, although IBS is not life-threatening, it creates a large burden on global healthcare and causes a serious reduction in the quality of life^2^. However, a therapeutic protocol for the disease, including pharmacological therapy, has not been established. Four subtypes of IBS are recognized, depending on the predominant stool pattern: IBS with constipation (IBS-C), IBS with diarrhea (IBS-D), mixed IBS (IBS-M) and un-subtyped IBS^3^.

Although the mechanism underlying the pathogenesis of IBS is not completely understood, several contributory factors have been proposed, including brain-gut axis dysregulation, enhanced visceral perception, altered intestinal microbiota, post-infectious changes in gastrointestinal function and enhanced immunologic reactivity^4^,^5^,^6^,^7^,^8^. Given that no single causal trigger for IBS has been identified, a combination of physiologic, genetic, environmental and psychological factors seems to be responsible for the visceral hypersensitivity and altered bowel conditions observed in IBS patients. In particular, mental stress in early childhood (such as the loss of a parent, neglect or abuse) is known to induce IBS-related phenotypes in both humans and animals^9^,^10^.

Previously, the pharmacological treatment of IBS-D involved classic anti-diarrheal agents, such as loperamide and anticholinergic drugs. Some clinical studies have also suggested the effectiveness of antidepressants, although others reported contradictory results^11^. Recently, alosetron and ramosetron, two serotonin 3 (5-HT_3_) receptor antagonists, were approved for patients with IBS-D^12^,^13^. This is based on the fact that inhibition of 5-HT_3_ receptors in the intestine is associated with the suppression of its motility and fluid secretion^12^. Rifaximin, an antibacterial drug, and eluxadoline, which has both μ-opioid receptor agonist and δ-opioid receptor antagonist activity, were also recently approved for IBS-D^14^,^15^. However, thus far, the outcomes of pharmacological therapy for IBS-D are unsatisfactory^16^. Furthermore, as the 5-HT_3_ receptor also regulates other physiological functions, the use of 5-HT_3_ receptor antagonists is clinically restricted due to adverse effects, such as ischemic colitis^17^. In fact, the use of alosetron for IBS-D patients is permitted only when no alternative therapies are available^17^. Thus, new target proteins for IBS-D drugs, which enable long-term treatment without serious adverse effects, need to be identified^16^,^18^. One potential approach is to phenotypically screen compounds for their ability to reduce visceral hypersensitivity and stress-induced defecation in animals.

The number of drugs reaching the marketplace each year is decreasing, mainly due to the fact that unexpected adverse effects of potential drugs are revealed in clinical trials. Thus, we have proposed a new strategy for drug discovery and development (drug re-positioning), which focuses on the use of existing medicines for alternative indications^19^. This strategy screens compounds with clinically beneficial pharmacological activity from a library of medicines that are already in clinical use to develop them for new indications. The advantage of this strategy is the decreased risk of unexpected adverse effects in humans because the safety aspects of these drugs have already been well characterized^19^. Furthermore, as the library size of approved medicines is relatively small, the phenotypic screening of compounds in animals is much easier to implement using a drug re-positioning strategy rather than a general drug discovery approach.

Aminophylline (a mixture of theophylline and ethylenediamine in a 2:1 molecular ratio) is traditionally used as a bronchodilator^20^,^21^. Although the molecular mechanism governing its efficacy has not been fully defined, aminophylline (theophylline) has been reported to have both antagonizing activity for adenosine receptors (ARs) and inhibitory activity on phosphodiesterases (PDEs), both of which are believed to mediate the bronchodilatory activity of aminophylline^22^,^23^. Among the four major subtypes of AR (A_1_ARs, A_2A_ARs, A_2B_ARs and A_3_ARs), aminophylline (theophylline) is an antagonist of A_1_ARs, A_2A_ARs and A_2B_ARs but not of A_3_ARs^24^,^25^. A_1_ARs are mainly expressed in the brain and spinal cord, while A_2A_ARs are expressed in the brain, spinal cord and peripheral tissues/cells (such as the spleen, thymus, leucocytes, small intestine, and colon)^26^,^27^. A_2B_ARs are mainly expressed in the peripheral tissues, such as the large intestine^28^. Various pathophysiological roles of ARs have been reported, and agonists and antagonists for these receptors have attracted considerable attention as drugs for various diseases^26^. PDE inhibitors also have various pharmacological activities, and some have already been approved for clinical use^29^.

Although previous studies have reported both positive and negative effects of adenosine on intestinal motility and nociception^30^,^31^,^32^, the role of each AR subtype in IBS-D remains unknown. In an animal model of acute somatic pain (hot-plate test), an antagonist of A_2B_ARs but not of A_1_ARs or A_2A_ARs showed an analgesic effect^31^, whereas in another animal model of somatic pain (formalin test), an A_2A_AR antagonist acted as an analgesic^30^,^33^. A_2B_AR-knockout mice have been reported to exhibit decreased stool frequency^32^ whereas an A_2B_AR antagonist enhanced colonic contraction in rats^30^. Further, activation of A_1_ARs in the spinal cord has an analgesic effect^34^. On the other hand, it has been reported that inhibition of PDE4 suppresses stress-induced defecation^35^. These results suggest that aminophylline (theophylline) may affect visceral hypersensitivity and stress-induced defecation in IBS-D patients and animal models either positively or negatively; however, no study to date has investigated these effects.

In the present study, we used an *in vivo* phenotype screening and drug re-positioning strategy to search for candidate drugs for IBS-D. We screened a compound library consisting of clinically available drugs for the ability of the drugs to prevent stress-induced defecation and visceral pain, and identified aminophylline as a potential candidate. Analysis with a specific inhibitor for each subtype of PDE and AR suggested that PDE4 and A_2B_ARs probably mediated the inhibitory effect of aminophylline on stress-induced defecation. On the other hand, A_2A_ARs and A_2B_ARs appear to be involved in its inhibitory effect on visceral hypersensitivity. On the basis of these results, we propose that aminophylline may be a candidate drug for IBS-D.

## Results

### Effect of aminophylline on wrap restraint stress (WRS)-induced fecal pellet output

We selected 209 clinically used drugs, including bronchodilators, anticonvulsants, antibiotics, anti-hypertensives and anti-allergy drugs. We did not select anti-cancer drugs. The drugs were screened for their ability to suppress both the visceral pain response to repeated colorectal distension (CRD), and WRS-induced fecal pellet output in rats. Then, aminophylline was identified on the basis of its inhibition of both the visceromotor response (VMR) to CRD and WRS-induced fecal pellet output, as well as the available clinical data of its tolerability. We excluded positive drugs that are positive hits in the screening assay but had severe side effects, such as hypotension and hypoglycemia.

**Figure 1:**
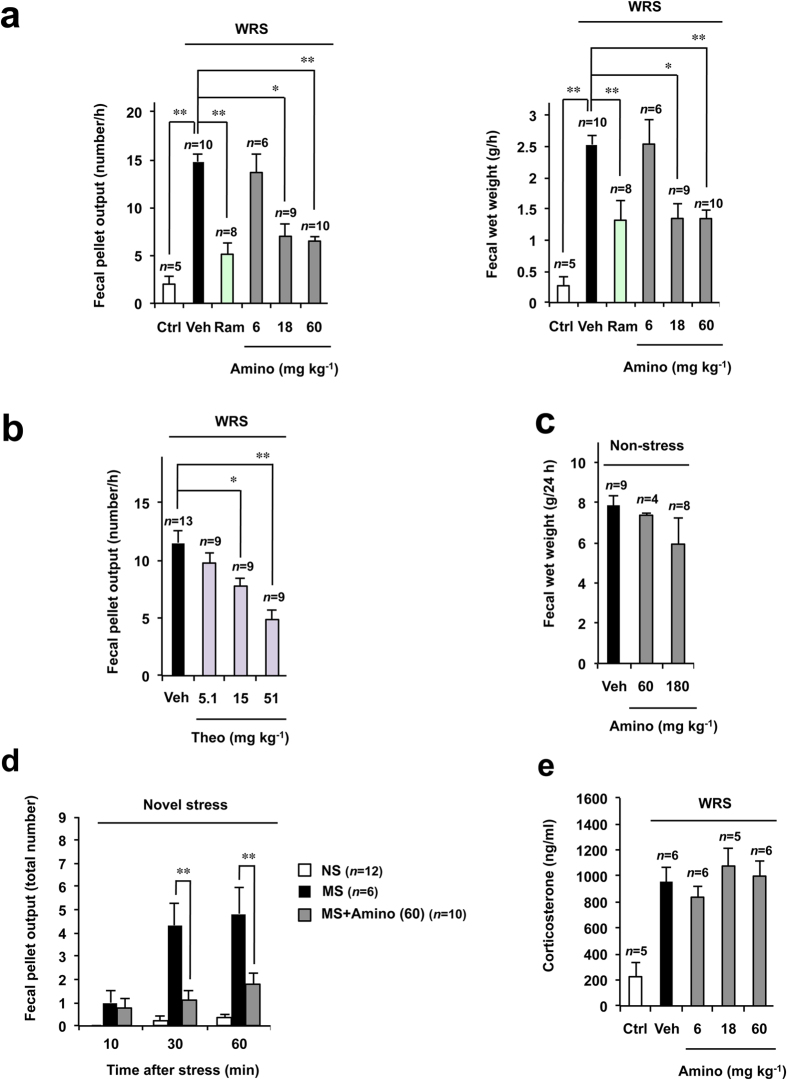
Effects of aminophylline on WRS- and novel stress-induced defecation in rats. Rats were subjected to maternal separation (MS) or non-maternal separation (NS) as described in the Materials and Methods (**d**). The rats received the indicated oral dose of aminophylline (Amino) (mg kg^−1^) (**a**,**c**–**e**), ramosetron (Ram) (0.03 mg kg^−1^) (**a**), theophylline (Theo) (mg kg^−1^) (**b**) or vehicle (Veh: saline) (**a**–**e**). Two hours after the administration, rats were exposed to WRS for 1 h (**a**,**b**,**e**), or novel stress (transfer to a new cage) (**d**) or remained undisturbed for 24 h (**c**). The number (**a**,**b**,**d**) and wet weight (**a**,**c**) of the fecal pellets excreted in 1 h (**a**,**b**), 24 h (**c**) or until the indicated time period (**d**) were determined. After the WRS, the plasma level of corticosterone was measured by ELISA (**e**). The values are the mean ± s.e.m. ^*^P < 0.05; ^**^P < 0.01 (Tukey test).

We first examined the effect of oral administration of aminophylline on WRS-induced fecal pellet output. As shown in Fig. 1a, rats subjected to WRS displayed an increase in the number and wet weight of fecal pellets compared to that in unrestrained control rats, as described previously^36^. Oral pre-administration of aminophylline (18 or 60 mg kg^−1^) significantly decreased both these indices in a dose-dependent manner (Fig. 1a). Oral pre-administration of ramosetron produced a similar effect (Fig. 1a).

Aminophylline is a complex of theophylline and ethylenediamine, and we found that oral pre-administration of theophylline also significantly decreased the fecal pellet output in rats subjected to WRS (Fig. 1b) at a dose equivalent to that of aminophylline (with respect to the molecular weight of theophylline). In contrast, as shown in Fig. 1c, aminophylline did not affect the fecal pellet output in rats that were not subjected to WRS, even at the higher dose of 180 mg kg^−1^. These results suggest that aminophylline can suppress WRS-induced defecation without affecting normal defecation.

It has been reported that neonatal rats subjected to maternal separation show higher sensitivity to a novel stress stimulus, which can be monitored by an increase in fecal pellet output^10^. We therefore examined the effect of aminophylline in this animal model. Indeed, as shown in Fig. 1d, maternal separation stimulated novel stress-induced fecal pellet output, a response that was suppressed by aminophylline.

We then examined the effect of aminophylline on the serum level of corticosterone after exposure to WRS. As shown in Fig. 1e, rats subjected to WRS exhibited a significant increase in their serum corticosterone level, an effect that was not ameliorated by pre-administration of aminophylline. This suggests that aminophylline affects WRS-induced fecal pellet output independent of the serum level of corticosterone.

### The mechanism undelying aminophylline-dependent suppression of WRS-induced fecal pellet output

**Figure 2:**
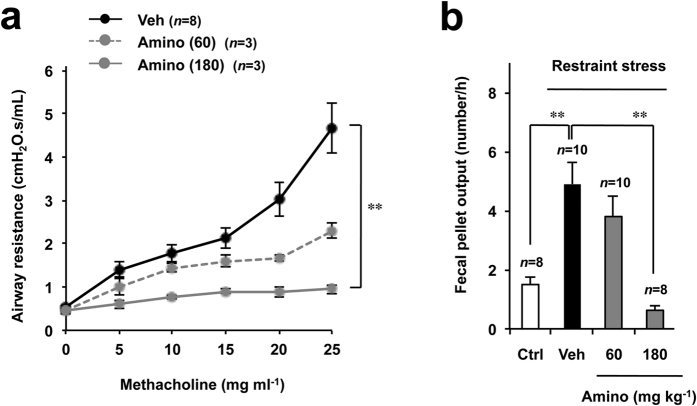
The relationship between the inhibitory effect of aminophylline on defecation and the bronchodilatory effect of aminophylline in mice. The indicated dose (mg kg^−1^) of aminophylline (Amino) (mg kg^−1^) or vehicle (Veh: saline) was orally administered to mice (**a**,**b**). After 1 h, the mice were exposed to nebulized methacholine for 5 times, and the airway resistance was determined after each methacholine challenge (**a**). Two hours after aminophylline administration, the mice were subjected to RS for 1 h and the number of fecal pellets excreted during the RS period (1 h) was determined (**b**). Control mice (Ctrl) were left to move freely in their cage. The values are the mean ± s.e.m. ^*^P < 0.05; ^**^P < 0.01 (Tukey test).

For both the clinical application of aminophylline for IBS-D patients and the identification of the molecular mechanism underlying aminophylline-dependent suppression of WRS-induced fecal pellet output, it is important to examine whether this novel pharmacological effect of aminophylline (suppression of defecation) is achieved at a dose similar to that required for its original pharmacological activity (bronchodilation). We therefore compared the dose-response profiles of aminophylline in terms of its inhibitory effect on defecation and its bronchodilatory effect. Given that we had already established the assay system for bronchodilation in mice^37^, we used mice for this comparative analysis. Significant inhibition of methacholine-induced bronchoconstriction (an increase in airway resistance; Fig. 2a) and significant inhibition of restraint stress (RS)-induced fecal pellet output (Fig. 2b) were observed after oral administration of 180 mg kg^−1^ aminophylline. The dose of aminophylline required for its bronchodilation also inhibited stress-induced defecation.

**Figure 3:**
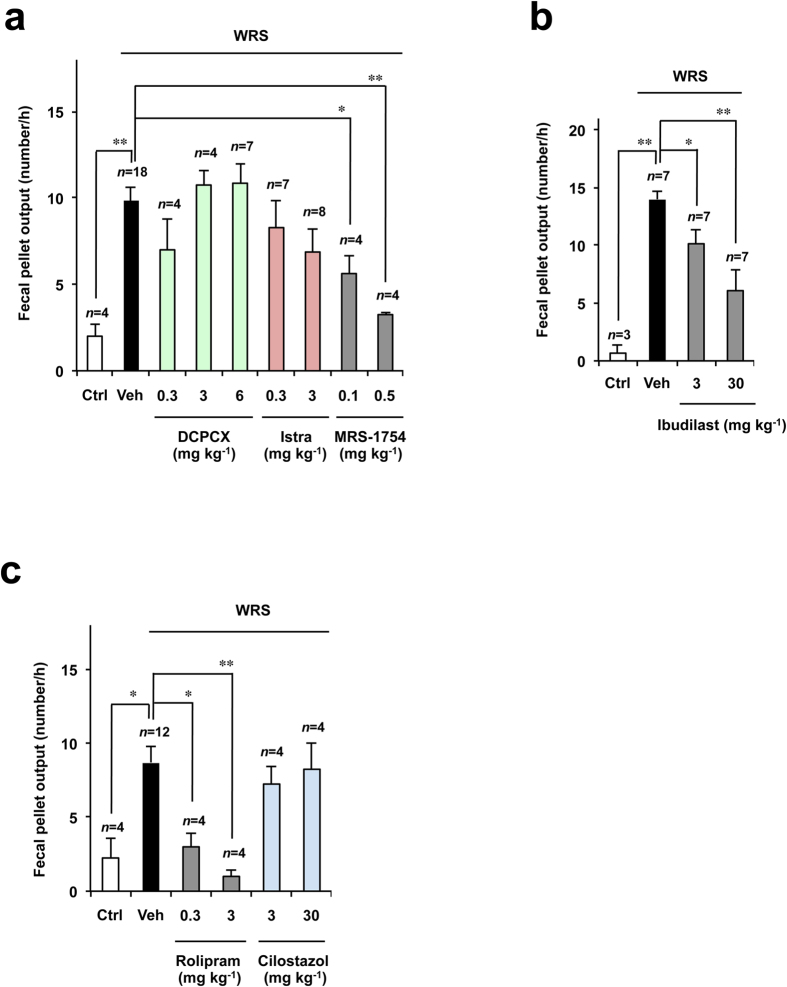
Effects of AR antagonists and PDE inhibitors on WRS-induced defecation in rats. Rats were intraperitoneally administered the indicated dose of DCPCX (a selective A_1_AR antagonist) (**a**), istradefylline (Istra) (a selective A_2A_AR antagonist) (**a**), MRS-1754 (a selective A_2B_AR antagonist) (**a**), ibudilast (a subtype non-selective PDE inhibitor) (**b**), rolipram (a selective PDE4 inhibitor) (**c**), cilostazol (a selective PDE3 inhibitor) (**c**) or vehicle (Veh: 1% methylcellulose) (**a**–**c**). Fifteen minutes after the administration, rats were subjected to WRS for 1 h. Control rats (Ctrl) were left to move freely in their cage. The number of fecal pellets excreted during this period (1 h) was counted. The values are the mean ± s.e.m. ^*^P < 0.05; ^**^P < 0.01 (Tukey test).

Therefore, we focused on the inhibitory effect of aminophylline on both PDEs and ARs. We first examined the effect of a specific antagonist of each AR subtype on WRS-induced defecation. Pre-administration of MRS-1754 (a subtype-specific antagonist of A_2B_ARs) significantly suppressed fecal pellet output in rats subjected to WRS in a dose-dependent manner (Fig. 3a). However, DPCPX and istradefylline (subtype-specific antagonists of A_1_ARs and A_2A_ARs, respectively) had no significant effect hereon (Fig. 3a).

We also examined the effect of PDE inhibitors on WRS-induced defecation. As shown in Fig. 3b, ibudilast (a subtype non-specific inhibitor of PDEs) significantly suppressed fecal pellet output in rats subjected to WRS. We then used a specific inhibitor of each subtype of PDE. As shown in Fig. 3c, rolipram (a subtype-specific inhibitor of PDE4) but not cilostazol (a subtype-specific inhibitor of PDE3) suppressed the WRS-induced fecal pellet output in rats. These results suggest that the inhibitory effect of aminophylline on WRS-induced defecation is probably mediated by its inhibitory effect on both A_2B_ARs and PDE4.

### Effect of aminophylline on visceral hypersensitivity to CRD

**Figure 4:**
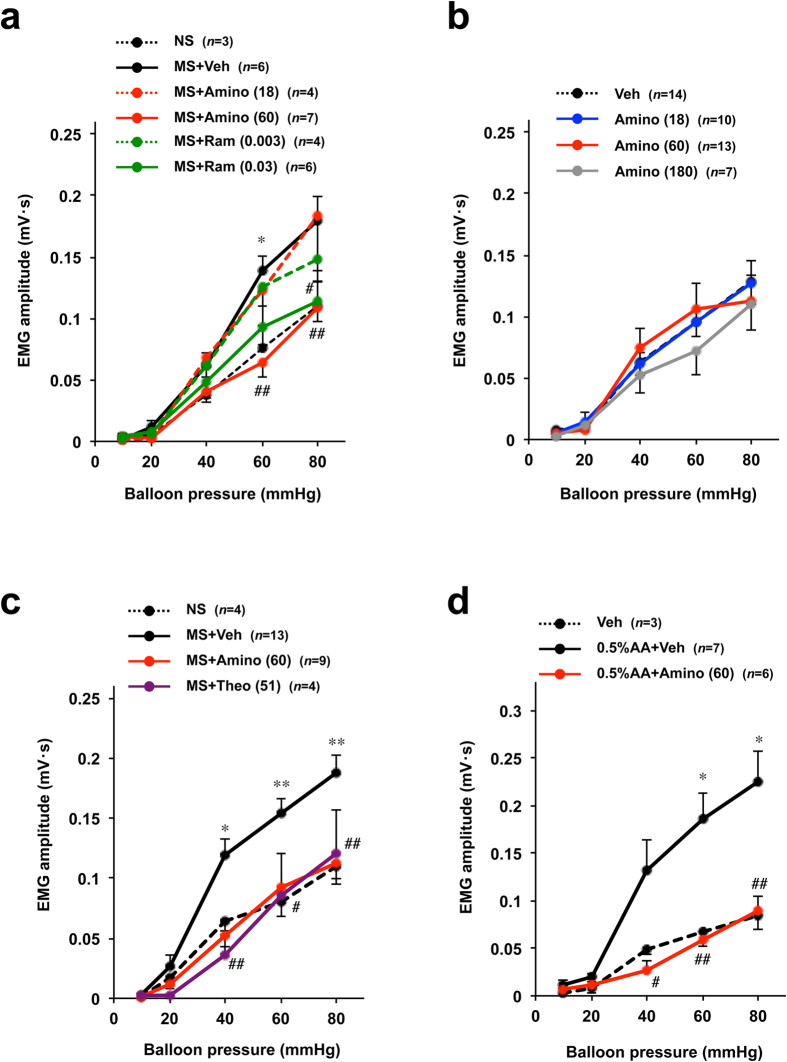
Effects of aminophylline on maternal separation- and acetic acid administration-induced visceral hypersensitivity to CRD in rats. Rats were subjected to maternal separation (MS) or administration of 0.5% acetic acid (AA) as described in the Materials and Methods. The MS and non-maternally separated (NS) rats were orally administered the indicated dose of aminophylline (Amino) (mg kg^−1^) (**a**,**b**), ramosetron (Ram) (mg kg^−1^) (**a**), theophylline (Theo) (mg kg^−1^) (**b**) or vehicle (Veh: saline) (**a**–**c**). Two hours later, the VMR to CRD was monitored by measuring the EMG. The values are the mean ± s.e.m. ^*^ or ^#^*P* < 0.05; ^**^ or ^##^*P* < 0.01 (^*^, vs NS; ^#^, vs Veh) (Tukey test).

To assess the effect of aminophylline on visceral hypersensitivity, we used a rat model of maternal separation-induced visceral hypersensitivity to CRD, one of the animal models of IBS^10^. As shown in Fig. 4a, the VMR evoked by CRD (EMG amplitude) increased according to the increase in balloon pressure, an effect that was stimulated in rats subjected to early maternal separation as described previously^10^. However, oral pre-administration of aminophylline (60 mg kg^−1^) significantly suppressed the VMR to a level similar to that observed in non-maternally separated rats (control rats) (Fig. 4a). Oral pre-administration of ramosetron produced a similar effect (Fig. 4a). On the other hand, pre-administration of aminophylline did not affect the VMR to CRD in control rats (without maternal separation) even at the higher dose of 180 mg kg^−1^ (Fig. 4b). We also found that administration of theophylline suppressed the VMR to CRD in rats subjected to maternal separation at a dose equivalent to that of aminophylline (with respect to the molecular weight of theophylline) (Fig. 4c). These results suggest that aminophylline and theophylline suppress the maternal separation-induced visceral hypersensitivity to CRD.

We then evaluated the therapeutic potential of aminophylline in an acetic acid-induced visceral hypersensitivity. In this model, rat pups received intracolonic administration of acetic acid at 10 days of age and visceral hypersensitivity to CRD was assessed at 5–6 weeks of age. As shown in Fig. 4d, rats subjected to the acetic acid treatment showed visceral hypersensitivity to CRD. However, pre-administration of aminophylline returned the sensitivity to the level observed in control rats.

### The mechanism underlying aminophylline-dependent suppression of visceral hypersensitivity to CRD

**Figure 5:**
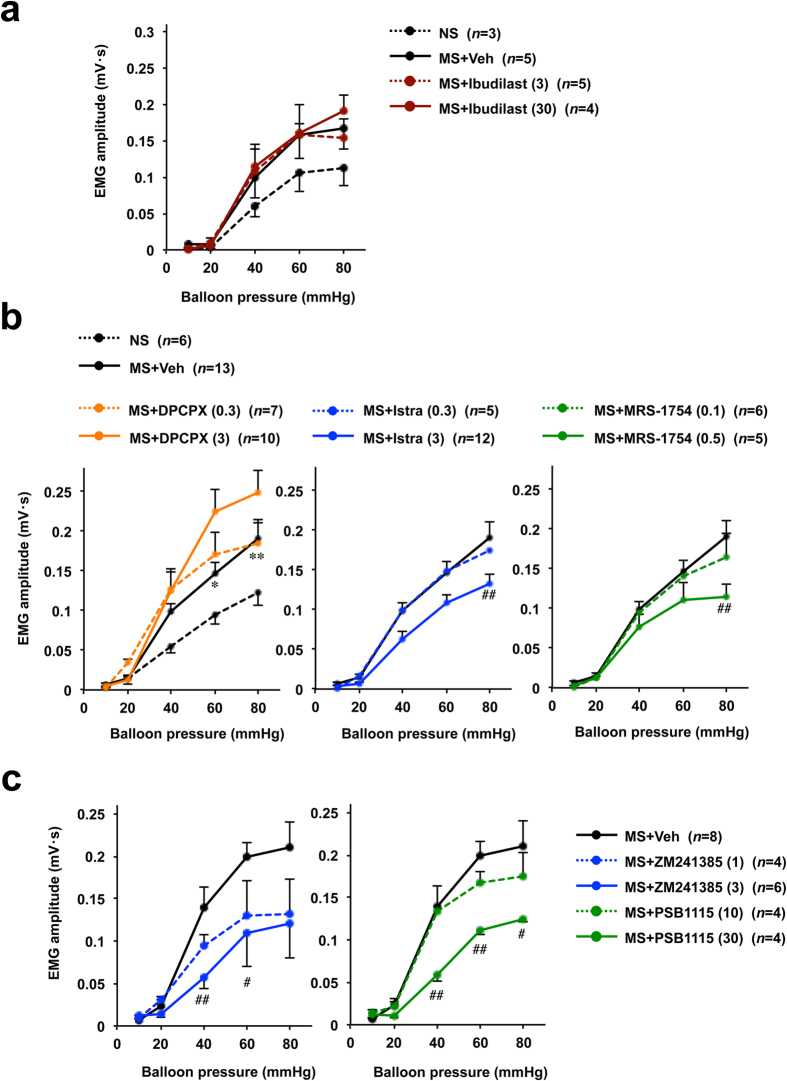
Effects of AR antagonists and PDE inhibitors on maternal separation-induced visceral hypersensitivity to CRD in rats. Rats subjected to maternal separation (MS) (**a**–**c**) or non-separation (NS) (**a**,**b**) were intraperitoneally administered the indicated dose (mg kg^−1^) of ibudilast (a subtype non-selective PDE inhibitor) (**a**), DCPCX (a selective A_1_AR antagonist) (**b**), istradefylline (Istra) (a selective A_2A_AR antagonist) (**b**), MRS-1754 (a selective A_2B_AR antagonist) (**b**), ZM241385 (a selective A_2A_AR antagonist) (**c**), PSB1115 (a selective A_2B_AR antagonist) (**c**) or vehicle (Veh: 1% methylcellulose) (**a**–**c**). Fifteen minutes later, the VMR to CRD was monitored. The values are the mean ± s.e.m. ^*^ or ^#^*P* < 0.05; ^**^ or ^##^*P* < 0.01 (^*^, vs Ctrl; ^#^, vs Veh) (Tukey test). The data for the MS + Veh in the three panels in (**b**) and the two panels in (**c**) are the same.

As shown in Fig. 5a, ibudilast did not significantly affect the VMR to CRD in rats subjected to maternal separation, suggesting that the inhibitory effect of aminophylline on visceral hypersensitivity to CRD is not mediated by inhibition of PDEs. Thus, we focused on ARs. Pre-administration of istradefylline or MRS-1754 significantly suppressed the VMR to CRD in rats subjected to maternal separation to an extent similar to that observed with aminophylline (Fig. 5b). In contrast, DPCPX did not suppress the VMR to CRD, and indeed a higher dose of this drug had a stimulatory effect (Fig. 5b). These results suggest that the inhibitory effect of aminophylline on maternal separation-induced visceral hypersensitivity to CRD is mediated by its inhibitory effect on both A_2A_ARs and A_2B_ARs. To confirm this hypothesis, we used another subtype-specific antagonist of A_2A_ARs and A_2B_ARs. As shown in Fig. 5c, both ZM241385 and PSB1115 (a subtype-specific antagonist of A_2A_ARs and A_2B_ARs, respectively) significantly suppressed the VMR to CRD in rats subjected to maternal separation.

**Figure 6:**
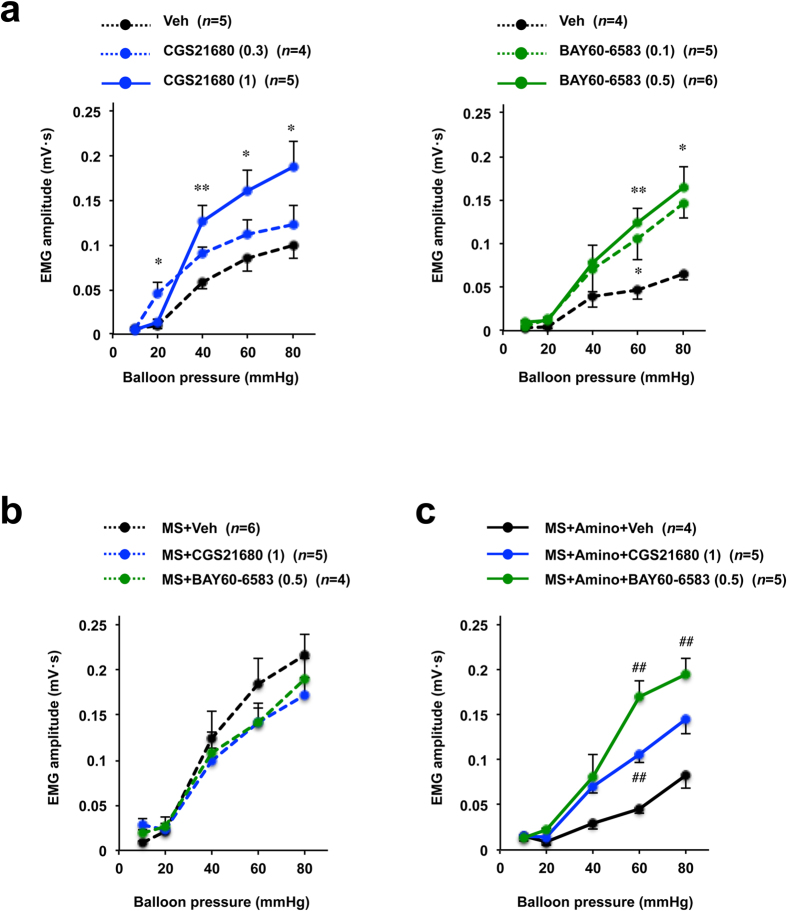
Effects of AR agonists on visceral sensitivity to CRD in control and maternally separated rats. Control rats (**a**) and maternally separated (MS) (**b**,**c**) rats were intraperitoneally administered the indicated dose (mg kg^−1^) of CGS21680 (a selective A_2A_AR agonist) (**a**–**c**), BAY60-6583 (a selective A_2B_AR agonist) (**a**–**c**), or vehicle (Veh: 1% methylcellulose) (**a**–**c**). Fifteen minutes later, the VMR to CRD was monitored. A 60 mg/kg of aminophylline (Amino) was orally administered to rats 2 h before the CRD test (**c**). The values are the mean ± s.e.m. ^*^ or ^#^*P* < 0.05; ^**^ or ^##^*P* < 0.01 (^*^, vs Veh; ^#^, vs MS + Amino + Veh) (Tukey test).

To investigate the involvement of A_2A_AR and A_2B_AR activation in the development of visceral hypersensitivity to CRD, we examined the effect of CGS21680 (a subtype-specific A_2A_AR agonist) and BAY60-6583 (a subtype-specific A_2B_AR agonist) on VMR to CRD in normal rats. We found that both CGS21680 and BAY60-6583 produced a stimulatory effect on VMR to CRD in normal rats (Fig. 6a). We also assessed the effect of CGS21680 and BAY60-6583 on the rats subjected to maternal separation, and found neither agonist affected the VMR to CRD in maternally separated rats (Fig. 6b). Finally, we examined whether an A_2A_AR or A_2B_AR agonist could inhibit the ameliorative effect of aminophylline on maternal separation-induced visceral hypersensitivity to CRD. Both CGS21680 and BAY60-6583 significantly stimulated the VMR to CRD in maternally separated rats pretreated with aminophylline (Fig. 6c).

## Discussion

Although various types of drugs are prescribed for IBS-D patients and several target proteins for IBS-D drugs have been proposed, an appropriate pharmacological therapy has not yet been established. Furthermore, the adverse effects of the current IBS-D drugs restrict the clinical use of these drugs (see introduction). Thus, novel drugs that target novel proteins and enable long-term treatment without serious adverse effects are required. Here we adopted a phenotypic screening approach and a drug re-positioning strategy to search for suitable drug candidates. Specifically, we screened for compounds that could suppress both the VMR to CRD and stress-induced defecation in rats, and identified aminophylline as a promising candidate. Aminophylline or theophylline has been clinically used as a bronchodilator and both its antagonizing activity on ARs and its inhibitory activity on PDEs have been reported^23^. However, thus far, the potential beneficial effects of these drugs on IBS have not been proven in pre-clinical or clinical studies.

Here, the oral administration of aminophylline prevented not only WRS-induced defecation but also novel stress-induced defecation in rats and RS-induced defecation in mice. However, in rats, aminophylline did not affect defecation under normal conditions and the serum level of corticosterone under stress conditions, suggesting that aminophylline specifically prevented stress-induced defecation independent of the function of the hypothalamic-pituitary-adrenal axis. The lack of an effect on normal defecation would appear to be beneficial for its clinical application in IBS patients. The dose-response profiles of aminophylline in terms of its inhibitory effect on fecal pellet output and its bronchodilatory effect were similar in mice. Theophylline also showed an inhibitory effect on WRS-induced fecal pellet output. These results suggest that the inhibitory effect of aminophylline on stress-induced defecation is mediated by a mechanism similar to that for its bronchodilatory effect. In other words, by antagonizing ARs and/or inhibiting PDEs. We showed that pre-administration of a subtype-specific antagonist of A_2B_ARs but not of A_1_ARs or A_2A_ARs significantly suppressed WRS-induced fecal pellet output in rats. We also demonstrated that a subtype non-specific inhibitor of PDE and a subtype-specific inhibitor of PDE4 but not PDE3 had a similar effect. These results suggest that the inhibitory effect of aminophylline on WRS-induced fecal pellet output is mediated by its inhibitory effect on both PDE4 and A_2B_ARs. However, no direct evidence for this effect was presented in this study. Therefore, confirmation that a selective PDE4 activator or a selective A_2B_AR agonist can inhibit the ameliorating effect of aminophylline is warranted. Furthermore, these results are consistent with those of previous reports showing that A_2B_AR-knockout mice have decreased stool frequency^32^ and that inhibition of PDE4 suppresses stress-induced defecation^35^. Nevertheless, they do not agree with the finding by Antonioli *et al*. that A_2B_AR antagonists enhance colonic contraction in rats^30^. Thus, our findings are in contrast to those of Antonioli *et al*. We believe that the results of Antonioli *et al*. may not necessarily reflect the overall effect on colonic propulsive activity *in vivo* because their experiments were tested *in vitro* using a longitudinal smooth muscle strip. On the other hand, Chandrasekharan *et al*. reported that A_2B_AR knockout mice and A_2B_AR antagonist-treated mice showed delayed colonic emptying, decreased stool retention and decreased stool frequency^32^. The authors also suggested that these phenotypes were mediated by inhibition of colonic circular muscle relaxation through the blockade of A_2B_ARs, which are involved in NO release from enteric neurons. We postulate that such a mechanism may underlie our results.

Oral administration of aminophylline or theophylline prevented maternal separation- and acetic acid administration-induced visceral hypersensitivity to CRD but did not affect the visceral sensitivity under normal conditions. The inability of these drugs to affect normal visceral sensitivity may be beneficial for their clinical application in IBS patients. Regarding the mechanism governing the inhibitory effect of aminophylline on visceral hypersensitivity to CRD, we showed that a subtype non-specific inhibitor of PDE did not significantly affect the hypersensitivity response. In contrast, pre-administration of subtype-specific antagonists of A_2A_ARs and A_2B_ARs but not A_1_ARs significantly suppressed the VMR to CRD in rats subjected to maternal separation. These results suggest that the inhibitory effect of aminophylline on maternal separation-induced visceral hypersensitivity to CRD is mediated by its inhibitory effect on both A_2A_ARs and A_2B_ARs. We confirmed this hypothesis using additional subtype-specific A_2A_AR and A_2B_AR antagonists (ZM241385 and PSB1115, respectively) and subtype-specific A_2A_AR and A_2B_AR agonists (CGS21680 and BAY60-6583, respectively) in the presence of aminophylline. Given that it is known that PSB1115 does not cross the blood-brain barrier^38^, this result suggests that A_2B_ARs expressed on peripheral tissues (such as the large intestine) rather than those expressed in the central nervous system mediate the inhibitory effect of aminophylline on visceral hypersensitivity. Furthermore, we found that A_2A_AR and A_2B_AR subtype-specific agonists stimulated visceral sensitivity to CRD in normal rats but not in maternally separated rats. We speculate that A_2A_R and A_2B_R receptor sensitivity is decreased in maternally separated rats because these receptors are already bound by adenosine. Thus, we also infer that the occurrence (or enhancement) of a tonic adenosine release during stress rather than an increased expression of A_2A_AR and A_2B_AR is involved in the development of visceral hypersensitivity to CRD in stressed rats. As described in the introduction, previous studies have shown that A_2A_AR and A_2B_AR antagonists produce an analgesic effect in an animal model of somatic pain^31^. However, to the best of our knowledge, our study is the first to demonstrate the inhibitory effect of A_2A_AR and A_2B_AR antagonists on visceral pain and hypersensitivity. On the other hand, we found that administration of a higher dose of an A_1_AR antagonist stimulated the VMR to CRD, which was consistent with the finding of a previous study showing that the activation of A_1_ARs produced an analgesic effect^34^.

We propose that aminophylline and theophylline are candidate drugs for IBS-D due to their ameliorating effects on both stress-induced defecation and visceral hypersensitivity. Their ability to suppress maternal separation-induced visceral hypersensitivity to CRD is particularly important, given that most current IBS-D drugs, except for 5-HT_3_ receptor antagonists, have not been reported to affect visceral hypersensitivity in this model. Furthermore, since the inhibitory effect of aminophylline on WRS-induced defecation appears to be mediated by a mechanism similar to that which underlies its bronchodilatory effect, aminophylline may show therapeutic efficacy at a dose used clinically and of which the safety has already been confirmed in humans.

Our results have also revealed a novel target protein class for IBS-D drugs: the A_2B_ARs. Specific antagonists for these receptors may therefore be beneficial for the treatment of IBS-D patients, although it is possible that aminophylline may still prove superior to such specific antagonists. We therefore propose that a pilot clinical study in which the efficacy of aminophylline in IBS-D patients is tested should be performed, given that this is already possible without the need of pre-clinical and phase 1 clinical studies.

## Methods

### Chemicals and animals

PSB1115 was purchased from Santa Cruz Biotechnology, Inc (Santa Cruz, CA). Methylcellulose, ibudilast and rolipram were obtained from Wako Pure Chemical Industries (Osaka, Japan). Medetomidine chloride (Domitor^®^) and butorphanol tartrate (Vetorphale^®^) were obtained from Meiji Seika Pharma Co., Ltd. (Tokyo, Japan) and midazolam was purchased from SANDOZ (Tokyo, Japan). Istradefylline and theophylline were purchased from Sigma (St. Louis, MO). Cilostazol and aminophylline were obtained from LKT laboratories, Inc. (St Paul, MN). MRS-1754 and BAY60-6583 were from Tocris Bioscience (Bristol, UK) and 1,3-dipropyl-8-cyclopentylxanthine (DCPCX), ZM241385 and CGS21680 were from Abcam (Cambridge, UK). ICR mice (5- or 6-week-old males, 28–33 g), primiparous late pregnant Wistar female rats and normal male Wistar rats (4- or 5-week-old, 150–250 g) were obtained from Charles River Laboratories Japan (Yokohama, Japan). The animals were housed under conditions of controlled temperature (22–24 °C) and illumination (12-h light cycle) conditions for 1 or 2 weeks before experiments. The experiments and procedures described here were performed in accordance with the Guide for the Care and Use of Laboratory Animals as adopted and promulgated by the National Institutes of Health, and were approved by the Animal Care Committees of Keio University and St. Marianna University.

### Measurement of VMR to CRD

The VMR to CRD was monitored as described^39^, with some modifications. Briefly, rats were deeply anesthetized with a mixture of medetomidine chloride (0.5 mg kg^−1^), midazolam (2.5 mg kg^−1^), and butorphanol tartrate (2.5 mg kg^−1^), and electromyography electrodes (Star Medical, Tokyo, Japan) were sutured into the external oblique muscle of the abdomen for electromyogram (EMG) recording. Electrode leads were tunneled subcutaneously and were exteriorized at the nape of the neck for future access. After the surgery, rats were housed individually and allowed to recuperate for 6 days before being used for measurement of the VMR. The rats were restrained in a plastic conical-shape tube (diameter, 6 cm; height, 15 cm), 15 min before the EMG recording. A polyethylene bag (length, 2 cm) was inserted in the distal colon, positioned 1 cm proximal to the rectum and connected to a balloon catheter. The pressure and volume of the balloon were controlled and monitored by a pressure controller-timing device (Distender Series II; G & J Electronics, Toronto, Canada), connected to the balloon. The rats were subjected to repeated CRD (12 times at 80 mm Hg; duration, 30 s; interstimulus interval, 300 s) for drug screening or to phasic CRD (10, 20, 40 60 and 80 mm Hg; duration, 20 s; interstimulus interval, 150 s) for the estimation of drug activity. Aminophylline or theophylline in saline or ramosetron in 1% methylcellulose were administered orally 2 h before CRD. The other drugs (AR antagonists, AR agonists and PDE inhibitors) were dissolved in 1% methylcellulose and were intraperitoneally administered 15 min before CRD. EMG data were collected and analyzed using the 8 STAR software package (version 6.0–19.2 for Windows; Star Medical, Tokyo, Japan). VMR evoked by contraction of the external oblique muscle was quantified by calculating the area under the curve (AUC) of the voltage alteration graph. The data were expressed as the result of the subtraction of the baseline VMR from the VMR during CRD (EMG amplitude). The baseline was consisted of the data collected 20 s before each CRD.

### Maternal separation- and acetic acid-induced colonic hypersensitivity to CRD

Neonatal maternal separation was performed as described previously^10^ with some modifications. Primiparous late pregnant Wistar female rats were individually housed for about a week prior to giving birth (10–15 pups/rat). The pups were separated from their dams for 3 h every day for 10 days (from postnatal day 2 to 12). Separations were conducted between 9 AM and 12 AM. The pups were placed in plastic cages that contained a heater pad (30 °C–33 °C), and were placed in a room separated from the dams. Non-maternally separated group (control pups) was left undisturbed with their dams. From postnatal day 12, both groups of rats were left undisturbed except for routine cage cleaning every two days. At 5–6 weeks of age, the VMR to CRD was examined in both groups of rats.

Acetic acid-induced colonic hypersensitivity was performed as described previously^40^ with some modifications. At 10 days of age, rat pups were subjected to intracolonic injection of 0.2 ml of 0.5% acetic acid in saline in a position 2 cm from the anus; control rats received an equal volume of saline. At 5–6 weeks of age, the VMR to CRD in both groups of rats was measured.

### Stress-induced fecal pellet output

WRS-, RS-, and novel stress-induced fecal pellet output was monitored as described previously^36^,^39^, with some modifications.

To monitor the WRS-induced fecal pellet output in rats, the rats received an oral dose of aminophylline or theophylline in saline (2 ml kg^−1^) or ramosetron in 1% methylcellulose (2 ml kg^−1^) 2 h before WRS. The other drugs (AR antagonists or PDE inhibitors) were dissolved in 1% methylcellulose and were injected intraperitoneally 15 min before WRS. Rats were subjected to WRS for 1 h and the number or wet weight of fecal pellets excreted during this period was determined. The wet weight of fecal pellets excreted during 24 hours was measured to evaluate the influence of aminophylline on defecation in non-stressed rats. WRS was performed as described previously^36^. Briefly, the rats were lightly anesthetized with isoflurane and their foreshoulders, upper forelimbs and thoracic trunk were wrapped in paper tape to restrict but not prevent movement. The animals recovered from isoflurane within 2–5 min and mobile immediately thereafter. The control rats were anesthetized with isoflurane but were not wrapped.

To monitor RS-induced fecal pellet output in mice, the mice were placed individually into a 50 ml Falcon tube (Becton Dickinson, Franklin Lakes, NJ) for 1 h. These tubes were small enough to restrain each mouse but were large enough to allow breathing. The control mice were left to move freely in their cage. Aminophylline in saline (10 ml kg^−1^) was orally administered 2 h before RS. The number of fecal pellets excreted during the RS period (1 h) was counted.

Control (non-maternally separated) and maternally separated rats (see above) were tested for responsiveness to a novel stress stimulus. This was induced by transferring the rats from their home cage with a white paper towel to a new cage with a wired mesh, as described previously^10^. Aminophylline was orally administered 2 h prior to exposure to a novel stress stimulus. The rats were placed in the new cage for 1 h, and the number of fecal pellets excreted during this period was counted.

### Measurement of lung airway resistance

Measurement of lung airway resistance was performed with a computer-controlled small animal ventilator (FlexiVent, SCIREQ, Montreal, Canada) as described previously^37^. The mice were anesthetized with chloral hydrate (500 mg kg^−1^), a tracheotomy was performed, and an 8-mm section of metallic tube was inserted into the trachea. Mice were mechanically ventilated at a rate of 150 breaths per min, using a tidal volume of 8.7 ml kg^−1^ and a positive end-expiratory pressure of 2–3 cm H_2_O. For measurement of the methacholine-induced increase in airway resistance, mice were exposed to nebulized methacholine (1 mg ml^−1^) five times for 20 s, with a 40 s interval, and airway resistance was measured after each methacholine challenge by the snap shot technique. Aminophylline was orally administered to mice 1 h before the test. All data were analyzed using the FlexiVent software (FlexiVent, SCIREQ, Montreal, Canada).

### Measurement of the plasma corticosterone level

After completion of the WRS experiment, the rats were euthanized and blood was collected to measure the plasma corticosterone level, using an ELISA kit (Cayman Chemical, Ann Arbor, MI), according to the manufacturer’s instructions.

### Statistical analysis

All values are expressed as the mean ± s.e.m. For the defecation experiments, one or two-way ANOVA followed by the Tukey test for unpaired results was used to evaluate differences between more than two groups or between two groups. For the bronchodilation and visceral hypersensitivity to CRD study, two-way ANOVA with repeated measures followed by the Tukey test was used. Differences were considered significant at *P* < 0.05.

## Additional Information

**How to cite this article**: Asano, T. *et al*. Aminophylline suppresses stress-induced visceral hypersensitivity and defecation in irritable bowel syndrome. *Sci. Rep.* **7**, 40214; doi: 10.1038/srep40214 (2017).

**Publisher's note:** Springer Nature remains neutral with regard to jurisdictional claims in published maps and institutional affiliations.
